# Mothers’ Experiences During the 2022 Infant Formula Shortage in Washington D.C.

**DOI:** 10.1007/s10995-023-03860-9

**Published:** 2023-12-26

**Authors:** Allison C. Sylvetsky, Sarah A. Hughes, Janae T. Kuttamperoor, Hailey R. Moore, Jeanne Murphy, Jennifer Sacheck, Emily R. Smith

**Affiliations:** 1 Department of Exercise and Nutrition Sciences, Milken Institute School of Public Health, The George Washington University, 950 New Hampshire Avenue NW, Washington, D.C. 20052 USA; 2 Division of Psychology, Children’s National Hospital, 111 Michigan Ave NW, Washington, D.C. 20010 USA; 3 School of Nursing, The George Washington University, 1919 Pennsylvania Ave. NW, Washington, D.C. 20006 USA; 4 Department of Global Health, Milken Institute School of Public Health, The George Washington University, 950 New Hampshire Avenue NW, Washington, D.C. 20052 USA

**Keywords:** Infant formula, Pediatrics, Mental health, Infant feeding

## Abstract

**Introduction:**

An unprecedented shortage of infant formula occurred in the United States (U.S.) in 2022 and posed widespread challenges to infant feeding nationwide. The purpose of this study is to investigate mothers’ experiences during the 2022 infant formula shortage and its perceived impacts on infants’ diet and health.

**Methods:**

Mothers (*n* = 45) of infants under 8 months old from Washington D.C. were invited to participate in a virtual study meeting during the summer of 2022. Mothers completed surveys regarding their demographics, infants’ anthropometrics, infant feeding practices, information they have received about infant feeding, and knowledge about infant feeding practices. They then participated in a qualitative interview about their experiences during the infant formula shortage.

**Results:**

Overarching themes were: the shortage (1) had adverse impacts on mothers’ mental and emotional health; (2) had significant financial and intangible costs; (3) led to changes in infant feeding practices; (4) social and family networks were helpful in navigating the shortage; and (5) mothers felt fortunate to have resources to breastfeed and/or obtain formula.

**Discussion:**

The infant formula shortage adversely impacted mothers’ mental and emotional health, and was costly, in terms of financial and intangible costs. Findings demonstrate the need to develop clinical and policy approaches to support mothers in feeding their infants and provide education about safe infant feeding practices.

**Supplementary Information:**

The online version contains supplementary material available at 10.1007/s10995-023-03860-9.

## Significance

An unprecedented shortage of infant formula occurred in the United States (U.S.) in 2022 and posed widespread challenges to infant feeding nationwide. Findings call attention to concerning impacts of the infant formula shortage on mothers’ mental and emotional health and highlight the disproportionate adverse impacts on Black mothers and mothers from low-income households. Enhanced efforts to connect mothers with resources such as community health workers are needed to support mothers in providing their infants with proper nutrition, particularly during shortages or other unexpected crises that may pose a barrier to infant feeding.

**Supplementary Information:**

The online version contains supplementary material available at 10.1007/s10995-023-03860-9.

## Introduction

An unprecedented shortage of infant formula occurred in the United States (U.S.) in 2022. This is alarming because the vast majority of U.S. infants are partially or entirely reliant on infant formula for nutrition, with only one in four infants exclusively breastfed for the first six months of life (United States Centers of Disease Control and Prevention, 2023b). Infants from low-income households are particularly likely to rely on infant formula, and 56% of all U.S. infant formula sold each year is consumed by infants enrolled in the WIC program (United States Department of Agriculture, 2022). Disproportionate reliance on infant formula among mothers enrolled in the WIC program is related to persistent racial/ethnic and socioeconomic disparities in breastfeeding in the U.S. (Jones et al., 2015), which result largely from social determinants including disparities in access to paid maternity leave, lactation support, and breastfeeding education (Standish & Parker, 2022).

The infant formula shortage was caused in part by voluntary recalls of several formulas produced by Abbott Nutrition, the shutdown of an Abbott Nutrition plant in Michigan in February 2022 due to bacterial contamination (United States Food and Drug Administration, 2023), and ongoing supply chain interruptions resulting from the COVID-19 pandemic (Calder et al., 2021). Because Abbott is among the largest suppliers of infant formula in the U.S. and produces specialized formulas for infants with severe allergies, gastrointestinal conditions, and metabolic disorders (Choi et al., 2020), access to specialized formulas rapidly diminished early in 2022. This resulted in a lack of appropriate nutrition for infants with these and other health conditions (Abrams & Duggan, 2022). The availability of routine formulas also declined drastically, posing challenges to infant feeding nationwide (Abrams & Duggan, 2022). By May 2022, out-of-stock rates were 74% nationally (Paris, 2022) and 82.4% in Washington, D.C. (“What You Need To Know About the Baby Formula Shortage,” 2022). The purpose of this qualitative study was to investigate mothers’ experiences during the infant formula shortage and examine perceived impacts of the shortage on infants’ diet and health. We also examined mothers’ sources of information about infant formula and their knowledge about the safety of alternative approaches for infant feeding during the shortage.

## Methods

### Recruitment and Sample

Mothers were recruited from Washington D.C. between June 2022 and August 2022 using community listservs and social media groups. Interested mothers were contacted by the research team to determine study eligibility. Inclusion criteria were (1) residing in Washington D.C. and (2) having a child less than 8 months old. The age range of up to eight months was selected to include both younger infants who were likely only consuming breast milk and/or formula as well as older infants who had been introduced to solid foods. This was intended to allow us to explore if mothers had increased the amount of or accelerated the introduction of complementary foods and/or cow’s milk relative to the recommended ages of 4–6 months and 12 months, respectively.

Mothers were recruited through convenience sampling and those who initially enrolled in the study were predominantly highly educated and non-Hispanic white. Therefore, eligibility for SNAP (Supplemental Nutrition Assistance Program) and/or WIC (Special Supplemental Program for Women, Infants, and Children) and/or identifying as Black/African American or Hispanic/Latino, were added as inclusion criterion in mid-July 2022 to enroll a more diverse sample of mothers. Neighborhood listservs and listservs of community organizations geared toward mothers and families in Wards 5, 7, and 8 of Washington, D.C., which are underserved areas, were used to target recruitment efforts to engage mothers from communities of color and/or underserved backgrounds.

### Procedures

Mothers were scheduled for a 30-minute, virtual, study meeting with a research team member in a private Zoom™ room. Mothers provided informed consent via RedCap™ prior to completing a brief survey. The survey included questions about the mother’s and infant’s demographics, infant’s birthweight and current weight, infant feeding practices, whether the mother had received advice from their doctor during the formula shortage; information sources influencing their decisions about infant feeding and what sources of information they trust (Appleton et al., 2020), and whether they perceived various alternatives to infant formula as safe. Following completion of the survey, an in-depth interview was conducted by one of two trained, female, interviewers using a semi-structured guide developed by the research team (Supplement 1). The guide included questions about mothers’ feelings related to the shortage, difficulties finding formula during the shortage, the extent to which mothers perceived the shortage impacted their infants’ diet, weight, and health, and how the shortage affected their experience as the mother of an infant.

Interviews occurred between June 22, 2022, and September 15, 2022, at which point forty-five mothers enrolled and saturation of the data had been reached. All interviews were recorded and transcribed verbatim using NVivo Transcription. Each mother received $50 as compensation via direct deposit, check, or debit card per participant preference. All study procedures were reviewed and approved by the Institutional Review Board at the George Washington University (IRB #: NCR224282) and reporting of qualitative findings is in accordance with the COREQ guidelines.

### Data Analysis

Descriptive statistics were used to summarize participants’ demographics and survey responses. Three coders independently coded an initial subset of three transcripts using Microsoft Word, which formed the basis of a shared codebook. Two coders coded the remaining transcripts using the shared codebook. The coders added new codes as they emerged and reorganized existing codes to develop the final codebook (Supplement 2). All transcripts were reviewed by the three coders to ensure coding complied with the final codebook, and discrepancies were resolved through discussion among the three coders and an additional research team member. After coding was completed on all transcripts, similarities and differences across race/ethnic and income subgroups (i.e., white vs. Black/African American and eligible vs. ineligible for SNAP or WIC) were examined using an exploratory approach by comparing the presence and frequency of codes across subgroups. Thematic analysis was used to identify emergent themes and subthemes, which were discussed amongst the research team, and refined collaboratively. Representative quotations for each theme and subtheme were selected.

## Results

### Participant Characteristics

The sample was racially diverse (Table 1), with 31% self-reporting as Black or African American, 11% self-reporting as Asian, and 7% self-reporting as more than one race. Of 11 mothers who reported SNAP and/or WIC eligibility, 9 (82%) self-identified as Black or African American. The mean age of the infants was 4.5 months and approximately half reported their infant was female (49% female, 51% male).

**Table 1:** Participant demographics and infant feeding practices, *n* = 45

	*N* (%)
**Race/Ethnicity**	
White	23 (51%)
Asian	5 (11%)
Black	14 (31%)
More than one race	3 (7%)
**Hispanic** ^**1**^	
No	41 (93%)
Yes	3 (7%)
**Educational Attainment**	
Did not complete high school	1 (2%)
Completed high school	7 (16%)
Completed college	13 (29%)
Completed graduate or doctorate degree	24 (53%)
**Receive WIC and/or SNAP Benefits**	
No	34 (76%)
Yes	11 (24%)
**Infant Age (mean ± SD), months**	4.5 ± 2.1
**Infant sex**	
Boy	23 (51%)
Girl	22 (49%)
**Infant Birthweight (mean ± SD), kilograms**	3.1 ± 0.7
**Infant weight (mean ± SD), kilograms**	6.4 ± 2.5
**Feeding Approach**	
Breast milk only	13 (29%)
Formula only	13 (29%)
Both breast milk and formula	19 (42%)
**Use of specialty formula**	
Yes	16 (36%)
No	29 (64%)
**Change in formula type due to shortage** ^**2**^	
Yes	14 (44%)
No	18 (56%)

Note: Some percentages do not sum to 100% due to rounding

^1^Based on *n* = 44 because one participant indicated that they preferred not to respond

^2^Based on *n* = 32 participants who reported feeding formula, whether exclusively or partially

### Quantitative Results

Most mothers reported using infant formula, whether exclusively (29%) or in combination with breast milk (42%), while 29% reported exclusively breastfeeding their infant. Of those using formula, 36% indicated using specialty formula, and nearly half (44%) reported switching brands and/or types of formula due to the shortage.

While 69% of mothers reported receiving information about infant feeding from their doctor or midwife (for their present infant), only 18% reported receiving feeding advice from their doctor or midwife specifically regarding the formula shortage (Table 2). Most mothers indicated family (58%) and healthcare workers (60%) influenced their opinion about what to feed their infant, and half indicated they trusted friends or family to provide accurate information about infant feeding. While 84% of mothers reported they trusted medical professionals to provide accurate information about infant feeding, along with medical organizations (e.g., American Academy of Pediatrics) and government agencies (e.g., United States Centers for Disease Control and Prevention), they did not feel supported in obtaining infant formula during the shortage. Additionally, although most mothers correctly indicated that making their own infant formula, substituting toddler milk for infant formula, or diluting infant formula were unsafe, several mothers (9%, 7%, and 9%, respectively) perceived these practices as safe. None of the mothers indicated cow’s milk as a safe alternative for infant formula.

**Table 2:** Mothers’ sources of information and beliefs about infant feeding practices and causes of the formula shortage, *n* = 45

Survey Question	*N* (%)
**Did you receive advice from your doctor or midwife about feeding your infant?** ***(N*** **, % yes)**	31 (69%)
**Did you receive advice from your doctor or midwife about feeding your infant specifically in light of the infant formula shortage? (N, % yes)**	8 (18%)
**Do any of the following influence your opinion about what to feed your baby?** ^**1**^	
Family or loved ones	26 (58%)
Healthcare workers	27 (60%)
Politicians	0 (0%)
Social media influencers	4 (9%)
Religious leaders	0 (0%)
None of the above	13 (29%)
**Which information sources do you trust to provide accurate information about infant feeding?** ^**1**^	
TV, radio, newspaper	7 (16%)
Social media	7 (16%)
Internet	7 (16%)
Friends or family	21 (47%)
Medical professionals	38 (84%)
Medical organizations	33 (73%)
Government agencies	27 (60%)
Religious leaders	0 (0%)
**Mothers were asked to indicate whether the following statements were true or false**:	
It is safe to make my own baby formula, % true	4 (9%)
Mixing water with evaporated milk is a good alternative to store-bought formula, % true	1 (2%)
Buying or trading breastmilk online is safer than switching to a new infant formula, % true	0 (0%)
Toddler ‘formula’ or toddler drinks are a good substitute for regular baby formula, % true	3 (7%)
Cow’s milk is a safe alternative for baby formula for infants, % true	0 (0%)
It is safe to switch between baby formulas, % false	8 (18%)
It is unsafe to ‘water down’ baby formula to make it last longer, % false	4 (9%)

^1^Percentages do not sum to 100% because participants could select multiple responses

### Qualitative Results

#### Theme 1. The Infant Formula Shortage Adversely Impacted Mothers’ Mental and Emotional Health

A key overarching theme was the infant formula shortage adversely impacted mothers’ mental and emotional health (Table 3). Mothers experienced significant anxiety related to the infant formula shortage, specifically surrounding the prospect of not being able to find formula and fear that other mothers were unable to locate formula. Mothers also reported anxiety about their infant gaining weight appropriately if they were unable to locate formula. They described feelings of sadness, shock, and disbelief that a formula shortage could happen in the U.S. Even mothers who were exclusively breastfeeding reported considerable anxiety about needing to rely on breastfeeding and not having formula as a back-up, given the possibility that formula may not be available. Mothers described feelings of general stress about the shortage, as well as anger about the situation. Guilt about feeding their infant formula and heightened pressure to breastfeed and maintain their milk supply were also described.

**Table 3:** The infant formula shortage adversely impacted mothers’ mental and emotional health

**Theme** Subtheme	Selected Relevant Quotations
**Stress**	Having an infant is stressful enough as it is and having to deal with formula shortages - it’s really, really scary. (White, not eligible for WIC or SNAP benefits) It [the shortage] has just made it significantly more stressful because you’re already stressed out by having a newborn, and not knowing if they’ll have food next week is kind of something you didn’t think you would have to worry about. (White, not eligible for WIC or SNAP benefits)
**Anger**	In this country at this time, the fact that we can say it is hard to safely feed your baby when clearly all they want us to do is crank out babies is mind boggling and infuriating, and just makes you want to burn things down. (White, not eligible for WIC or SNAP benefits) You shouldn’t have to worry about whether or not you can feed your baby. It’s just horrific. (White, not eligible for WIC or SNAP benefits)
**Shock**	It’s mind boggling that this is happening. (White, not eligible for WIC or SNAP benefits) I was like seriously? We just had COVID. Everything together. I was like another shortage this can’t be true. (White, not eligible for WIC or SNAP benefits)
**Sadness**	I left stores crying. (White, not eligible for WIC or SNAP benefits) I can’t even talk about I’m going to start crying. I just think it’s so sad and upsetting that, yeah, people aren’t able to feed their kids. (White, not eligible for WIC or SNAP benefits)
**Anxiety**	I don’t think anybody would have foreseen the sort of emotional distress that comes with worrying that you’re not going to be able to feed your baby. (White and Asian, not eligible for WIC or SNAP benefits) I’m scared. I am new mom. Everything is new, everything is scary, and to go through this. (White, not eligible for WIC or SNAP benefits)
Anxiety about finding formula	It’s a big source of stress, and I’ve tried to keep that anxiety in check. But at times, all of a sudden, it’s like, “holy crap, what if I can’t feed my baby?” That’s scary. (White, not eligible for WIC or SNAP benefits) The uncertainty of if I would have formula, when I can get it, how often I could find it, it was really stressful. (Black or African American, eligible for SNAP benefits)
Anxiety about finding specific type of formula	She was on Similac Sensitive, but I couldn’t find it. And I mean, I searched and searched and searched. I couldn’t find it…it made it harder because I was trying other milks and it just wasn’t working for her. (Black or African American, eligible for both WIC and SNAP benefits) It has been somewhat scary, especially because we have been asked to give my baby a formula without dairy. (White, not eligible for WIC or SNAP benefits)
Anxiety about others having difficulty obtaining formula	A big part of my stress was thinking about all the people who are in different situations and the impact that it would have on them…it’s just heartbreaking. (More than one race, not eligible for WIC or SNAP benefits) I feel very afraid. It was frightening to be a new mother myself, but also imagine all the other mothers who were already living through such a hard time, in terms of like everything going on in the world, the pandemic and everything, and then, you know, the fear of not being able to feed your baby is horrifying. (Black or African American, not eligible for WIC or SNAP benefits)
Fear about the prospect of having to use formula	It made me scared to think that that I might have to use formula. (White, not eligible for WIC or SNAP benefits) Because of the shortage I stay [breastfeeding] for three months until now… try my best because I’m afraid. (White, eligible for both WIC and SNAP benefits)
Anxiety about milk supply	But when it’s [formula] not available, all of a sudden it’s like, “OK, I need to get like breastfeeding supplements to try and produce more milk now and make sure that my baby’s fed.” (White, not eligible for WIC or SNAP benefits) It has made me more anxious about the supply of breast milk that I have and wanting to make sure that I have enough frozen so that it’ll be OK. (White, not eligible for WIC or SNAP benefits)
Anxiety about infant gaining appropriate weight	I don’t want to get to that point where I can’t find her milk and she start losing weight again. (Black or African American, eligible for both WIC and SNAP benefits) He was obviously losing weight, which every baby does, but I was super worried that he was never going to get any milk. (White, not eligible for WIC or SNAP benefits)
**Pressure to breastfeed**	I normally hate pumping and it’s not something I really want to do. But obviously right now, I just don’t really trust the formula situation… I just basically powered through a lot of the nipple pain and stuff like that. I was like, “I can’t mess up my breastfeeding right now.” (White, not eligible for WIC or SNAP benefits) I found myself pushing to breastfeed at all costs, essentially even when I really needed to take a nap and could have just used some formula. (White, not eligible for WIC or SNAP benefits)
Pressure to maintain milk supply	I really felt a lot of pressure to maintain breast milk supply because especially when it first started, there really was no end in sight. (Black or African American, not eligible for WIC or SNAP benefits) The nights are hard and I wish my husband would be able to take a shift so I could get more sleep…I don’t want to impact my supply. (White, not eligible for WIC or SNAP benefits)
**Guilt about feeding formula**	It made me feel guilty for stopping breastfeeding…I felt a lot of guilt about stopping breastfeeding and all these feelings of being a bad mother for not breastfeeding - they came back. (White and Asian, not eligible for WIC or SNAP benefits) I wish I would’ve stuck with breastfeeding. I wouldn’t have to go through this trying to figure out if my baby gonna have milk for the next two weeks or how one can is going to last because there’s no formula in the city. (Black or African American, eligible for WIC benefits)

#### Theme 2. The Infant Formula Shortage had Significant Financial and Intangible Costs

Another key overarching theme was that navigating the infant formula shortage had significant financial and intangible costs (Table 4). Mothers described financial strain resulting from the shortage; and in some cases, obtaining formula came at the expense of other basic household necessities, such as food or rent. Mothers who were enrolled in WIC described difficulties using their WIC benefits for formula during the shortage. Even mothers who indicated no difficulties affording formula described spending more on formula than planned due to rising costs of formula and the need to purchase formula in larger quantities or “stockpile.” Mothers also described intangible costs, including spending exorbitant amounts of time and energy searching for formula; this entailed driving from store to store, sometimes for hours, and searching online. Mothers also described feeling exhausted from the shortage, sacrificing their physical and emotional health, and changing their diet and lifestyle to prolong breastfeeding and/or continue pumping to avoid reliance on formula.

**Table 4:** The infant formula shortage had significant financial and intangible costs

**Theme** Subtheme	Selected Relevant Quotations
**Time**	Whatever our time costs to go out and run to five different stores. (White, not eligible for WIC or SNAP benefits) Imagine two days in my car at night, in the morning…I have to be early before the shortage, before the baby finishes the can. I must search in advance every day. (White, eligible for both WIC or SNAP benefits)
Time at stores	It was not at the big-name brand stores, like Safeway or Giant or Harris Teeter. I had to go to a neighborhood store that was across town. Just to find the milk. (Black or African American, eligible for both WIC and SNAP benefits) I must have driven around six CVS’s, three Whole Foods, every Target; all day I was driving around trying to find my particular brand, and I couldn’t. (White, not eligible for WIC or SNAP benefits)
Time using technology	I’m on formula groups on Facebook. I am calling or texting or sending emails to friends and family, asking them for help. They’re sending me texts, “Is this the right thing to get?” “How much do you want?” “Oh, I’m only able to get this much.” (White, not eligible for WIC or SNAP benefits) After each feeding in the middle of the night, I look online and see if I could find formula instead of going back to sleep. (More than one race, not eligible for WIC or SNAP benefits)
**Exhaustion**	I’m really tired from it [the shortage]. It’s very overwhelming trying [to find formula]. Right now, I’m down to one can and I have to go on a milk spree really soon. So, it’s kind of draining. It’s also kind of overwhelming, knowing that the last time I went out and I went to 10 plus stores before I was able to get one can of milk. (Black or African American, eligible for both WIC and SNAP benefits) I feel stress. Worry. I’m kind of tired of riding around looking for formula (Black or African American, eligible for WIC benefits)
**Costs of continued breastfeeding**	Because of the shortage, I was trying a lot harder, investing more in things to help with pumping… spending the time and money to try to do more breastfeeding and pumping. (Asian, not eligible for WIC or SNAP benefits) There’s nothing about breastfeeding that is free. It’s time intensive. You need supplies for pumping, you need new bras. (White, not eligible for WIC or SNAP benefits)
Physical costs	I don’t think I would have kept up with it [breastfeeding] knowing that I had a crutch, an alternative… there would have been nights where I’m like, “I’m exhausted, I just going to go to sleep. He can have a formula bottle in the morning.” (White, not eligible for WIC or SNAP benefits) Breastfeeding does change your body and it’s strenuous, so you rely on formula. But I didn’t feel like I had that option. (Black or African American, not eligible for WIC or SNAP benefits)
Emotional costs	Pumping is a chore. And it really sucks. And so, I was really looking forward to stopping. But when the formula shortage really was in its peak, we decided that it would be best to delay weaning, so that added weeks to my pumping journey. (White, not eligible for WIC or SNAP benefits) For my mental health and my ability to take care of my children, I had to stop pumping. But I also knew that things could get a little scary. And this [stopping breastfeeding] was going to potentially put my kid in jeopardy. (White, not eligible for WIC or SNAP benefits)
Time and energy costs	I was really struggling with pumping and was doing a lot of introspection on that. “Is it really worth pumping for an hour and a half to two hours a day just to get one little bottle of milk?” And I was finding myself surprisingly hesitant to give that up. (White, not eligible for WIC or SNAP benefits) I was going to a lot of lengths to make sure that I had enough breast milk for my baby. And if the infant formula shortage hadn’t have happened, I probably would have deviated towards more formula faster. (White, not eligible for WIC or SNAP benefits)
Costs to boost milk supply	I probably would not have worked this hard to make my breast milk come in if there would not have been a shortage. I spend the money getting different supplements…Lord knows, I’ve wasted hundreds of dollars trying different formulas, cookies, yeast. I don’t even know. Chiguru yeast. I try. Yeah, probably hundreds of things and some things worked and some things didn’t work. (Black or African American, not eligible for WIC or SNAP benefits) We’re not a wealthy household that we can afford a midwife or whatever…So, I was just talking to my family and they were just like “oh this worked for me” or “eat these kind of food and they’re going to get more milk” because the shortage was going on…and there were some teas I was taking, like maternity tea to help you with the milk. (White, not eligible for WIC or SNAP benefits)
**Cost of formula**	My husband bought these small cans and they were 30, 25 or 30, dollars. So, probably 10 dollars more than they should cost; it’s absurd that it costs what it costs in America. (White, not eligible for WIC or SNAP benefits) I remember my friend who told me about Bobbie [an infant formula subscription service]…I remember at the time being like, “Oh my gosh, that’s so expensive. That’s crazy.” But when the time came, there was no question. We have to do this. And the cost was not an issue anymore. (White, not eligible for WIC or SNAP benefits)
Financial strain	Usually, I budget to buy formula weekly based on my paycheck. But then [due to the shortage], I went to buy formula one week for six, seven weeks, which is a really big investment, all at once, simply so I wouldn’t run out. (White, not eligible for WIC or SNAP benefits) We have to actually spend money or food stamps on this milk. So, it’s hard to have the milk shortage right now. (Black or African American, eligible for both WIC and SNAP benefits)
Inability to use WIC vouchers	I actually get WIC, but I’m not able to use it because you can only use it in the area you live. You actually can’t use your WIC outside of your area. (Black or African American, eligible for WIC benefits) The old WIC program, it used to be when you used the voucher, they gave you five cans on the voucher. You have to find five. But if you don’t, I only bought three [because there was not more available], the other two cans are gone now because I already used the voucher for the three cans. So, I will have to come out of pocket for the other two cans. (Black or African American, eligible for both WIC and SNAP benefits)
Tradeoff between formula and other basic household needs	I have to take away from my [other] children because I have to buy formula with my food stamps. So, it takes away from the other kids. You know. That leaves me with looking at other places to get help with getting food in the house because I have spent the food stamps and my money trying to get formula. Yeah, it’s hard. (Black or African American, eligible for both WIC and SNAP benefits) For me, the baby is more important than rent and insurance. (White, eligible for both WIC and SNAP benefits)

#### Theme 3. The Infant Formula Shortage Led to Changes in Infants’ Diet

A third overarching theme was that the shortage led to changes in infants’ diets (Table 5). Mothers explained that their infant received fewer calories due to not having enough formula, resulting in they perceived to be inadequate weight gain. Mothers who were able to breastfeed mentioned freezing extra milk to have a back-up, and in some cases, donating extra breast milk to help others. Mothers also reported making efforts to conserve formula by reducing how much they provided to their infant, minimizing wasted formula, saving leftover formula, encouraging their infant to finish bottles, and earlier introduction of solid foods. Two mothers reported providing their infant with toddler milk or juice because infant formula was unavailable.

**Table 5:** The infant formula shortage led to changes in infants’ diet

Theme Subtheme	Selected Relevant Quotations
**Infant received fewer calories**	We’ve been adding a half a scoop of formula to his bottles. We kind of stopped when we were running low…I don’t know if those extra calories really would have made a difference…but he’s not gaining weight fast enough. (White or Caucasian, not eligible for WIC or SNAP benefits) She shouldn’t be on a diet as a newborn. I see a lot of babies that be looking all chubby and stuff, but my baby, I don’t get to see that with her due, due to the shortage. (Black or African American, eligible for both WIC and SNAP benefits)
**Feeding breast milk instead of formula**	It makes me want to breastfeed more, knowing that it might be a problem with me finding any milk. (Black or African American, not eligible for WIC or SNAP benefits) It’s like, “let’s not become dependent on something that may or may not be there for us,” and so I think that that has meant that instead of supplementing at times we just continue to breastfeed. (White or Caucasian, not eligible for WIC or SNAP benefits)
Freezing more milk	We are freezing a lot more milk…and I’m pumping at work so that we can make sure that when I’m not at home, we have milk to rely on and not formula. (White or Caucasian, not eligible for WIC or SNAP benefits) I breastfeed too, so I have a substitute if I were to just be absolutely out of milk. And I also have breast milk stored in the freezer as a backup. (Black or African American, eligible for both WIC and SNAP benefits)
**Stockpiling formula**	I think we got about 10 containers worth. And each container is good for a couple of weeks, so that’s almost half a year’s worth of supply at the rate he was drinking at the time. (Asian, not eligible for WIC or SNAP benefits) I’m acutely aware that you can’t run down the store down the street and just pick it up. You have to have a stockpile. (White or Caucasian, not eligible for WIC or SNAP benefits)
**Conserving formula**	[Prior to the shortage], I would make extra milk in case she would want it, and now I’m super regulated; I’m very protective of that scoop, try to get her on a schedule, no wasted formula, like we can’t afford to waste because it’s so scary. (White or Caucasian, not eligible for WIC or SNAP benefits) We have to be careful about how much we give her because we can’t just keep pouring out four ounces. (White and Asian, not eligible for WIC or SNAP benefits)
Minimizing wasted formula	Definitely not wanting to waste milk in a bottle; you know, making sure that he’s finishing bottles, and it’s frustrating if he doesn’t. (White or Caucasian, not eligible for WIC or SNAP benefits) I am really concerned about waste. And if she doesn’t eat the bottles, is the milk going to go bad? Because if it is, there is a scarcity around the formula, and it can’t be wasted. (White or Caucasian, not eligible for WIC or SNAP benefits)
Saving leftover formula	I probably stretched some of the limits: “there’s this much left, okay, we’re going to stretch it and go a little bit.” I’ve never gone more than, let’s say, two days, but I probably stretch some of the recommended limits. (White or Caucasian, not eligible for WIC or SNAP benefits) I try not to waste no milk, at all. I don’t care if it’s just this much in a bottle. I save it. She’s OK because that’s what we have. (Black or African American, eligible for both WIC and SNAP benefits)
**Donating milk**	I’ve actually been donating milk and tried to donate a little more with the formula shortage happening. I’ve tried to do a little extra pumping; just kind of knowing that the shortage is happening and trying in a small way to help out. (White or Caucasian, not eligible for WIC or SNAP benefits) I’ll probably start donating breast milk, which I wouldn’t necessarily have even really thought about if there were not this shortage happening. (White or Caucasian, not eligible for WIC or SNAP benefits)
**Switching type of formula**	I’m kind of hesitant to switch back and forth, but I also don’t really have a choice I would need to give her something. (White or Caucasian, not eligible for WIC or SNAP benefits) The biggest impact was really switching from ready-to-feed to the powder. (White or Caucasian, not eligible for WIC or SNAP benefits)
Hesitancy to switch formula	I just didn’t feel comfortable switching. So, we just kept searching until we found some [of the infant’s usual formula]. (Black or African American, eligible for SNAP benefits) I don’t think it’s healthy to keep changing the baby’s milk every few days or every week. (Black or African American, eligible for both WIC and SNAP benefits)
Gastrointestinal problems after switching type of formula	He ended up pooping, an incredible amount, nine times a day for several days. So, the impact is our son was fed, but he wasn’t fed the formula that he would normally eat. (White or Caucasian, not eligible for WIC or SNAP benefits) She had diarrhea, she was throwing up, and it was like the watery, lose stools….so it [the formula shortage] impacted her because she was sick [from having to switch formulas]. (Black or African American, eligible for both WIC and SNAP benefits)
**Earlier introduction of solid foods**	I am probably pushing solids a little bit more than I would be normally, just because it would be great to not be so reliant on formula and get off it quicker than I might have otherwise. (White or Caucasian, not eligible for WIC or SNAP benefits) I think we would have probably waited [to introduce solid food] until he could have handled things a little bit more and start using softer foods instead of going with all the purees and everything like that. (White or Caucasian, not eligible for WIC or SNAP benefits)

#### Theme 4. Social Networks Were Helpful in Navigating the Infant Formula Shortage but Mothers did not Feel Supported by the Government

Another theme was mothers found social networks helpful in locating infant formula but did not feel supported by the government and were frustrated by a general lack of support for breastfeeding (Table 6). Mothers explained they were able to locate formula through social media groups and friends and family in other parts of the country. A new or amplified sense of community was also described, where mothers were unified due to the difficulty of the situation.

**Table 6:** Social networks were helpful in navigating the infant formula shortage but mothers did not feel supported by the government

Theme Subtheme	Selected Relevant Quotations
**Support from social networks**	I sent a text saying, this is what my daughter is drinking, and if you see it, please grab it and just let me know. So, like my sisters, my friends, my children’s godparents, neighbors, pretty much everybody [was helping]. (Black or African American, eligible for SNAP benefits) As soon as we started to get low on the ready-to-feed, we reached out to my network. I reached out to my friends. I reached out to my co-workers. I reached out to my family and asked them. And that creates a web up and down the East Coast and across the country. (White, not eligible for WIC or SNAP benefits)
Social media groups	I’m seeing a lot of folks coming out to support each other, like in all these parent groups. I just see so many people posting, “I have extra, do you need this?” Everyone kind of comes together to support each other because of this. (Asian, not eligible for WIC or SNAP benefits) I’ve been relying on moms’ groups like on WhatsApp to get the formula that I need or do just yeah. And like groups of around the neighborhood where they give formula that they don’t no longer need kind of like to find the right brand that I need. (White, not eligible for WIC or SNAP benefits)
Family	My mother and sister are in South Carolina. My mother’s retired and my sister doesn’t have a traditional office job…once they learned about it [the shortage], they went on a scavenger hunt and were able to get it [our supply of formula] back up. (Asian, not eligible for WIC or SNAP benefits) It’s been really difficult because some stores, like the ones in my neighborhood usually don’t have it. As soon as it’s there, its gone. So it’s been really difficult and I’ve had to have my sister go further out in Maryland to try to find it. (Black or African American, eligible for SNAP benefits)
**Lack of government support**	It started to make the national news after it was already a big problem. And…it’s still a big problem. And, the news cycle has moved on. So, I think it’s just left parents to continue to try and figure it out themselves. (White, not eligible for WIC or SNAP benefits) It’s kind of like we don’t have a government, since the system is set up to step up in this type of moment. (White, not eligible for WIC or SNAP benefits)
Lack of policy solutions	It’s frustrating that we’re not seeing policy solutions. (White, not eligible for WIC or SNAP benefits) The politics and laws are not super supportive of moms. (White, not eligible for WIC or SNAP benefits)
Failure to prioritize needs of mothers and infants	The needs of infants and their caregivers are shunted to one side, except when it’s a convenient political football. (White, not eligible for WIC or SNAP benefits) We should be able to put our resources behind it to be able to take care of babies. That should be a priority. But it’s not a priority that’s getting fixed. (White, not eligible for WIC or SNAP benefits)
**General lack of support for breastfeeding**	I think of breastfeeding and when that didn’t go well, largely in part because of the poor lactation services. I think the breastfeeding would have eventually gone better if the care had been better, including the lactation services, and then I wouldn’t have needed to have gone home with infant formula. (More than one race, not eligible for WIC or SNAP benefits) There are all of these additional stressors involved with it [breastfeeding]. And in a world like we live in today, where you know, both parents have to work, daycare costs a million dollars. There should be alternatives for moms… (White, not eligible for WIC or SNAP benefits)

#### Theme 5. Mothers Felt Lucky to have the Ability to Breastfeed and/or Obtain Formula During the Shortage

A final emergent theme was mothers felt lucky to be able to breastfeed and have resources to obtain formula (Table 7). Breastfeeding mothers explained feeling fortunate that their infant was able to latch and/or that their milk supply was plentiful. Mothers reported feeling grateful that they had supportive family members and friends who helped them find formula. Mothers also described feeling grateful that they had resources to obtain formula, financial means to purchase more expensive formula or enroll in formula subscription services, and lived near a plethora of retail outlets selling formula. This sentiment was primarily described by white mothers. While white and Asian mothers often described using subscription, delivery, and text services to obtain formula during the shortage, such services were not mentioned by any Black or SNAP- or WIC-eligible participants.

**Table 7:** Mothers felt lucky to have the ability to breastfeed and/or obtain formula during the shortage

Theme Subtheme	Selected Relevant Quotations
**Ability to breastfeed**	It’s made me feel more grateful that I am able to breastfeed. (More than one race, not eligible for WIC or SNAP benefits) My body and my baby are cooperating, and it would be a different situation if they weren’t. (White, not eligible for WIC or SNAP benefits)
**Ability to obtain formula**	I would say that it has not affected my ability to feed my child directly, but only because of fluke circumstances, like lucky circumstances. (Black or African American, not eligible for WIC or SNAP benefits) I know that I have relative affluence and I have a big network, so I thought I would be OK because generally, that’s the truth. (White, not eligible for WIC or SNAP benefits)
Social resources	I feel lucky that I have the privilege to be able to source formula if I end up needing it from my neighbors. (White, not eligible for WIC or SNAP benefits) We were fortunate that we’ve had a lot of people with older children give us formula that was still in safety range and not expired or anything. (Black or African American, not eligible for WIC or SNAP benefits)
Financial resources	I guess I’ve been lucky enough that I can afford to order from Europe and that they don’t have a shortage there, so you just order online and it arrives two weeks. (White, not eligible for WIC or SNAP benefits) We had to get a different brand of formula that was approximately twice as expensive. Because we are fortunate enough to be able to afford it. (White and Asian, not eligible for WIC or SNAP benefits)
Access to subscription services	We signed up for a subscription service, so we feel a bit more secure with where we’re getting our formula from. (Asian, not eligible for WIC or SNAP benefits) We started ordering Bobbie [subscription] formula; and around the same time, like March or April, they put a pause on accepting new customers so that they could preserve their inventory for existing customers. And once I saw that, it took me like a couple of weeks to be like, “OK, I feel confident we’re still getting the formula we need.” (White, not eligible for WIC or SNAP benefits)
Built environment	I feel grateful that I was able to get like a six, seven-week supply because I scavenger hunt the city. (White, not eligible for WIC or SNAP benefits) I’m so sorry for the ladies who live in the countryside…they are worse off than us. (White, eligible for both WIC and SNAP benefits)

### Subthemes Across Sociodemographic Subgroups

Several other subthemes also differed across sociodemographic subgroups. While feeling heightened pressure to breastfeed and guilt about not breastfeeding were widely described by white and Asian mothers and those not eligible for SNAP or WIC, this was not frequently described by Black mothers or by mothers eligible for SNAP or WIC. Meanwhile, Black mothers and those eligible for SNAP or WIC tended to describe a hesitancy to switch formulas and indicated their infant required a specific formula due to a pre-existing condition. While receiving help finding formula from friends and family and social media networks were described across race and income subgroups, these resources were described more frequently by white mothers and those not receiving SNAP or WIC benefits.

## Discussion

The infant formula shortage adversely impacted mothers’ mental and emotional health; and, navigating the situation had significant and unanticipated financial, physical, emotional, and lifestyle-related (e.g., time) costs. Although in some cases mothers reported their infant received fewer calories due to the shortage and/or had gastrointestinal problems resulting from switching formula, most described altering their planned and/or preferred feeding practices to ensure their infant received adequate nutrition and did not suffer serious health consequences.

Unfavorable mental, emotional, physical, financial, and lifestyle-related impacts of the shortage were widely described irrespective of mothers’ sociodemographic characteristics; however, the most severe financial impacts were described by mothers who were eligible for SNAP and/or WIC. While WIC is a supplemental program and is not designed to meet all formula needs for infants enrolled in the program, mothers described challenges using their WIC benefits during the shortage and had to pay for formula out-of-pocket, resulting in significant financial strain. It is important to note that while the present study was conducted among mothers in Washington, D.C., resources and support available to families enrolled in WIC during the infant formula shortage varied across states, and complications using WIC during the shortage were observed throughout the U.S. (Kalaitzandonakes et al., 2023).

Mothers in our study expressed frustration at the general lack of support for breastfeeding and the physical, emotional, and time-related barriers to continue breastfeeding, consistent with prior literature (Li et al., 2008). In order to better support the unique needs of breastfeeding mothers, community-based organizations could be leveraged to connect mothers to resources, such as culturally relevant breastfeeding advertisements, resource guides, and virtual breastfeeding workshops (Lilleston et al., 2015). While feeling grateful to be able to breastfeed was described across race and income subgroups, Black mothers and mothers from low-income households described facing heightened challenges to breastfeeding during the shortage, including inflexible work schedules, shorter paid maternity leaves, and/or not working in an environment conducive to pumping. The U.S. remains the only industrialized country without a national paid maternity leave policy despite notable mental and physical health benefits for mothers and infants. And racial inequities in the conduciveness of the work environment for breastfeeding (Van Niel et al., 2020) are well-described barriers to breastfeeding in the infant feeding literature (Jones et al., 2015).

Adverse consequences of the formula shortage on mothers’ mental and emotional health are particularly alarming because the postpartum period is already a time of heightened stress (Tully et al., 2017). In the year following the birth of a child, 15% of women suffer from postpartum anxiety (Shorey et al., 2018), and approximately 12.5% of women in the U.S. have postpartum depression (United States Centers for Disease Control and Prevention, 2022). Both conditions have unfavorable short and long-term impacts on maternal and infant health (Grace et al., 2003) and also interfere with mother-child bonding (Dubber et al., 2015) and infant feeding outcomes (Fallon et al., 2016). Even in the absence of postpartum anxiety and/or depression, the months following childbirth involve sleep exhaustion, feelings of isolation, new demands of parenting, and changes in physique and sexuality, all of which detract from mothers’ mental and physical health (Walker & Murry, 2022). The COVID-19 pandemic has further exacerbated mental health challenges during the postpartum period and posed a barrier to accessing postpartum medical care and social support (Goldstein et al., 2022).

Difficulty finding formula and the prospect of hypothetically being unable to feed their infant were key contributors to reported stress and anxiety associated with the formula shortage. Even in the absence of an infant formula shortage and global pandemic, feelings of maternal shame and anxiety surrounding infant feeding practices (Thomson et al., 2015) and a lack of social and institutional support for breastfeeding in the U.S. (United States Centers of Disease Control and Prevention, 2023a) are well-documented. Experiencing breastfeeding problems leads to feelings of disappointment, guilt, failure, shame, and frustration (Ikonen et al., 2015), as breastfeeding is tied to mothers’ self-worth and identity (Demirci et al., 2018). Mothers in our study who reported exclusively breastfeeding described considerable anxiety about their milk supply and ability to avoid switching to formula due to the shortage. Stress associated with the perception of inadequate milk supply is widespread, and often leads to cessation of breastfeeding (Li et al., 2008) and/or supplementation with infant formula (Bookhart et al., 2021).

Mothers whose infants were fed formula described spending exorbitant amounts of time searching for formula, which further aggravated their stress and anxiety associated with the shortage. Time spent searching for formula was reported to contribute to exhaustion, disrupt work productivity and detract from time spent with older children. Considering that time constraints are a key barrier to healthy eating and physical activity among postpartum women (Ryan et al., 2022), which are important for preventing postpartum weight retention (Amorim Adegboye & Linne, 2013) and psychological morbidity (Dipietro et al., 2019), excessive time spent searching for formula may also have indirect, unfavorable impacts on mothers’ physical and psychological health.

Avoidance of infant health problems due to the formula shortage was predominantly attributed to mothers’ ability to breastfeed and/or ultimately finding sufficient formula after taking extreme or unconventional measures to acquire formula (e.g., leveraging social and family networks, searching multiple stores). While reported changes such as conserving formula through saving leftover formula for subsequent feeds and encouraging their infant to finish bottles did not result in major health problems, these practices are inconsistent with bottle-feeding guidance (United States Centers for Disease Control and Prevention, 2021) and may pose risks to infant food safety (United States Centers for Disease Control and Prevention, 2021) and contribute to overfeeding (Li et al., 2012). Furthermore, mothers reported providing their infant with solid foods earlier than planned, which is concerning because early introduction of solids is associated with later childhood obesity, particularly among formula-fed infants (Daniels et al., 2015). Two mothers reported providing their infant with bottles of toddler milk or fruit juice as a substitute when formula was unavailable during the shortage, both of which are not recommended by public health and medical organizations (Healthy Eating Research, 2019). Milk banks provide a safer alternative to high risk infant feeding practices; yet they remain underutilized, especially during public health emergencies, due to inaccessibility of donor milk, infant feeding needs (i.e., specialty formula), and mothers’ perceptions and preferences (Kalaitzandonakes et al., 2023).

Alarmingly, some mothers (7–9%) did not recognize that infant feeding practices such as making their own infant formula, substituting toddler milk for infant formula, or diluting formula to make it last longer were unsafe, which underscores the need for medical professionals and government agencies to provide mothers with more education about infant feeding. In fact, 5.6% of parents in the U.S. attempted to make formula at home and 13.3% substituted with a product other than formula or breast milk, such as cow’s milk (Kalaitzandonakes et al., 2023). Encouragingly, no mothers in the present study identified cow’s milk as a safe alternative to formula. As only 18% of mothers reported receiving information from their doctor or midwife about feeding their present infant (i.e., their most recent pregnancy and delivery as opposed to for any prior children) during the shortage, more proactive outreach by healthcare providers during emergencies is needed. Nearly half of the mothers in our sample reported that they trusted friends and family to provide accurate information about infant feeding, both of which are common sources of health misinformation (Suarez-Lledo & Alvarez-Galvez, 2021). Fortunately, healthcare providers and medical organizations were reported as the most trusted sources of information.

Social, partner, and family support were critical facilitators of acquiring infant formula during the shortage and are known to positively influence mothers’ physical and mental health during the postpartum period (Faleschini et al., 2019). Mothers described social media groups as a key source of support in navigating and locating formula during the shortage. Social media is an important source of social support during pregnancy and postpartum (Baker & Yang, 2018), and further efforts to provide postpartum women with support and reputable resources through online and in-person social networks are warranted.

### Limitations

Key limitations of this study include the focus specifically on mothers in Washington, D.C and the virtual data collection methods, which may limit the extent to which the findings are generalizable to mothers in other regions of the country or those that do not have access to internet and social media. Furthermore, only about one-quarter of the sample was eligible for WIC or SNAP, which is lower than national rates (United States Department of Agriculture, 2022) and 82% had educational attainment of college or higher, compared to approximately 38% nationwide (United States Census Bureau, 2022). While one of the two interviewers on the research team identifies as a woman of color, greater diversity among team members may have increased participation from marginalized communities. However, the consistency of the present findings with recent quantitative studies examining impacts of the infant formula shortage across the nation enhances the likelihood that our findings are broadly applicable. To our knowledge, this is the first study to examine mothers’ experiences during the 2022 infant formula shortage and is further strengthened by the racially diverse sample of participants.

## Conclusion

Findings call attention to concerning impacts of the infant formula shortage on mothers’ mental and emotional health and highlight the importance of social and family support in locating and acquiring infant formula during the shortage, which can be utilized in future emergency outreach efforts. These results demonstrate disproportionate adverse impacts of the shortage on Black mothers and mothers from low-income households and emphasize the urgent need to implement proper advocacy channels within the community to address systemic inequities in infant feeding practices. Enhanced efforts to connect mothers with community health workers and health advisors are needed to educate mothers about providing their infants with proper nutrition, particularly during shortages or other unexpected crises that may pose a barrier to infant feeding. Sustainable policy solutions to support infant feeding (i.e., national guaranteed paid maternity leave and increased access to milk banks), address this unprecedented crisis, and prevent recurrences, should be pressing public health priorities.

### Electronic Supplementary Material

Below is the link to the electronic supplementary material.

Supplementary Material 2

Supplementary Material 3

## Data Availability

Data described in the manuscript will be made available upon reasonable request to the corresponding author.

## Code Availability

The code book used to code the data manuscript will be made available upon reasonable request to the corresponding author.
