# Supplementary material for: Mothers’ Experiences During the 2022 Infant Formula Shortage in Washington D.C
Source: Matern Child Health J. 2023 Dec 26;28(5):873–86. doi: 10.1007/s10995-023-03860-9 (PMC11001681; doi:10.1007/s10995-023-03860-9)
Supplement: Supplementary file 1 — Supplementary Material 2 [file 10995_2023_3860_MOESM2_ESM.docx]

Infant Formula Interview Questions

1. **How do you feel about the current infant formula shortage?**
   1. Did the shortage change your feelings about feeding with formula?
2. **How has the current infant formula shortage impacted your ability to feed your baby?**
   - 1. Has it been difficult to find formula?
     2. Has it been difficult to afford formula?
     3. Has it been difficult to find formula that is suitable for your baby’s needs?
3. **Have there been changes to your baby’s diet resulting from the current infant formula shortage?**
   1. Has it impacted the amount of formula your baby eats?
   2. Has it impacted the type of formula your baby eats?
   3. Has it impacted how much solid food your baby eats?
   4. Has it impacted how much breast milk your baby eats?
4. **What types of challenges do you face feeding your infant and how have these challenges changed in light of the infant formula shortage?**
   1. How have you overcome these challenges?
5. **To what extent to you think that the current infant formula shortage has impacted your baby’s health?**
   1. Has it impacted your baby’s nutrition?
   2. Has it impacted your baby’s weight?
   3. Has it impacted your baby’s allergies or other pre-existing nutritional issues?
6. **What do you think caused the current infant formula shortage?**
7. **What advice or information have you received regarding feeding your infant and has this changed during the current infant formula shortage?**
8. **How has the infant formula shortage impacted your experience as a mother of an infant?**
9. **Is there anything else you would like to add about how the current infant formula shortage has impacted you or your baby?**
