# Supplementary material for: Mothers’ Experiences During the 2022 Infant Formula Shortage in Washington D.C
Source: Matern Child Health J. 2023 Dec 26;28(5):873–86. doi: 10.1007/s10995-023-03860-9 (PMC11001681; doi:10.1007/s10995-023-03860-9)
Supplement: Supplementary file 2 — Supplementary Material 3 [file 10995_2023_3860_MOESM3_ESM.doc]

**Infant Formula Finalized Code Book**

**Advice**

Advice Heard

Pump more (Joking advice)

(Or dangers of) Diluting baby's formula

Advise to prolong breastfeeding or start again

Babies can be given cow’s milk

Be careful of ratio of water to formula

Doctors advise to give/switch formula due to baby's health/challenge breastfeeding

Doctors advise to stick with one formula

Don't use Alimentum

Don't use evaporated milk

Introducing solids

Making formula at home

Normal dietary advice

Pro-breastfeeding advice

Lactation consultants give anti-formula advice/push for breastfeeding

Safe ways to boost breast milk supply

Search everywhere

Skeptical of advice

Stop breastfeeding whenever you're ready

Using goat milk

Which formula to use as substitute

Sources

Advice from lactation consultants

Advice from mom forums/ Facebook groups

CDC/ APA

D.C. Strong Start

Did not receive advice from pediatrician

Family giving advice

Hospital gives advice at birth

Leche League

Midwives/Doulas

No change in advice

Parent does their own research

Received advice from pediatrician/docs

The News

WIC gives advice

**Affordability**

Able to afford formula

Challenges of using WIC benefits for formula

Financial impact of formula shortage

Uses food stamps for formula

**Altered Behaviors due to shortage**

Adjusting lifestyle to prolong breastmilk

Began breastfeeding due to shortage

Can't switch formula because of baby

Donating breastmilk/formula

Feeding at daycare impacted due to shortage

Feels need to breastfeed/keep milk supply up

Friends pumping for them

Preserving and not wasting formula

Replacing infant formula with other beverages

Shortage (might force)/forces parents to switch formula types

Using only natural products now

Using unpreferred feeding method

**Breastfeeding Vs. Formula**

Baby has enough to eat due to breastfeeding and pumping

Breastfeeding and pumping won't be (wasn't at some point) enough and baby needs formula

Challenge to breastfeed/pump

Did not want to switch formulas

Not afraid to switch formulas

Parent wanted to breastfeed

Shortage changed feelings about formula

Shortage has not changed feelings about formula

Tries to buy more formula before running out

**Cause of Infant Formula Shortage**

Formula plant got shut down/babies sick

Formula recall contributed to shortage

Formula Shortage due to hoarding

People weren't working due to COVID/ Pandemic

Shortage caused by inadequate regulation or funding of FDA/ too many restrictions on foreign formula

Shortage caused by monopoly of services

Shortage caused by venture capital/ bureaucracy

Shortage due to premature babies formula recall and parents replacing with regular formula

Supply chain issue contributed to shortage/high demand

Unsure of what caused the shortage

WIC moms are buying other formula

**Formula Status**

Able to find formula (a particular formula)

Began stocking up on formula

Difficult to find formula (suitable for baby's needs)

Difficult to find specialized formula

European/ Other countries' Formula imported

Hospital/doctor gave formula

Ran out of milk/food

Stores are beginning to restock

Using subscription and delivery services for getting formula/ feels grateful for

Widespread/ time intensive search for formula

**Impact on baby's health/diet**

Baby has no pre-existing issues or changes in weight

Baby needs specialized formula due to pre-existing condition

Challenge getting baby to eat more

Challenges keeping baby fed

Change in baby's diet

Delayed solid food consumption

Discomfort due to switching formula brands

Increase in breastmilk consumption

Increase in formula consumption

Increase in solid food consumption

Less formula consumption

No difficulty feeding baby

Shortage accelerated/may accelerate introduction of solid foods

Shortage caused no change to baby's diet/able to feed baby

Shortage has not impacted baby's health

Baby's health impacted

Shortage has not impacted solid food intake yet

Unsure if shortage has impacted baby's health

**Mental health/ feelings**

Anxious/Stress

Anxiety about not finding formula/ having enough

Anxiety and stress about feeding baby

Anxious/stressed about not having formula as a backup

General Anxiety/Stress without a specific reason

Anxiety/frustration over recall/shortage

Prioritizing breastfeeding over mom's wellbeing/mental health

Stressed over breastmilk supply

Awareness brought to breastfeeding communities

Fed is best

Feels like a stronger mother due to shortage

Feels lucky to be able to breastfeed and pump

Feels lucky to be in a city

Feels needs of moms are not prioritized/system is not supportive

Feels sympathy/empathy for others dealing with formula shortage effects

Guilt about not breastfeeding/guilt to breastfeed

Hope for the end of the shortage

Shocked by how this could happen in the U.S./ current political environment

Shortage did not impact mental health very much

Shortage has increased bonding with child (maybe breastfeeding)

Shortage impacts ideal family size

Shortage on top of an already stressful time

**Sources of Support**

Facebook Groups/ Social Media/ Listservs

Family and Friends

General network of moms

Texting service for formula restocking

WIC/Mary's Center Ladyship
